# Effectiveness of mass treatment of *Schistosoma mansoni* infection in socially vulnerable areas of a state in northeastern Brazil, 2011–2014

**DOI:** 10.1186/s13690-021-00549-9

**Published:** 2021-03-09

**Authors:** Flávia Silvestre Outtes Wanderley, Ulisses Montarroyos, Cristine Bonfim, Carolina Cunha-Correia

**Affiliations:** 1 Department of Neurology, Faculdade de Ciências Médicas, Postgraduate Course on Health Sciences, University of Pernambuco, Rua Arnóbio Marques, 310, Santo Amaro, Recife, Pernambuco CEP 50100-130 Brazil; 2 Social Research Department, Joaquim Nabuco Foundation, Recife, PE Brazil

**Keywords:** Mass drug administration, *Schistosoma mansoni*, Social vulnerability

## Abstract

**Background:**

To assess the effectiveness of mass treatment of *Schistosoma mansoni* infection in socially vulnerable endemic areas in northeastern Brazil.

**Method:**

An ecological study was conducted, in which 118 localities in 30 municipalities in the state of Pernambuco were screened before 2011 and in 2014 (after mass treatment). Information on the endemic baseline index, mass treatment coverage, socio-environmental conditions and social vulnerability index were used in the multiple correspondence analysis. One hundred fourteen thousand nine hundred eighty-seven people in 118 locations were examined.

**Results:**

The first two dimensions of the multiple correspondence analysis represented 55.3% of the variability between locations. The human capital component of the social vulnerability index showed an association with the baseline endemicity index. There was a significant reduction in positivity for schistosomes. For two rounds, for every extra 1% of initial endemicity index, the fixed effect of 13.62% increased by 0.0003%, achieving at most 15.94%.

**Conclusions:**

The mass treatment intervention helped to reduce transmission of schistosomiasis in areas of high endemicity. Thus, it can be recommended that application of mass treatment should be accompanied by other control actions, such as basic sanitation, monitoring of intermediate vectors and case surveillance.

**Supplementary Information:**

The online version contains supplementary material available at 10.1186/s13690-021-00549-9.

## Background

Neglected tropical diseases (NTDs) have wide geographical distribution and affect millions of people worldwide, especially in the poorest and most vulnerable areas. Schistosomiasis is one of the most significant helminth infections in terms of public health. It is the second most prevalent parasitic disease among humans worldwide, is endemic in 54 countries and has been estimated to affect more than 2 billion people around the world [1–3]_._

Transmission of schistosomiasis is usually associated with biological, social, environmental and cultural factors. Suitable climatic and environmental conditions, together with inadequate domestic water supply, poor sanitation and poor hygiene conditions, are the main causes of persistent prevalence. Understanding the relationship between risk factors and schistosomiasis is important in implementing effective control programs [4].

*Schistosoma mansoni* infection is considered to be endemic in Brazil and is heavily affected by social and environmental conditions [5]. A recent national survey among schoolchildren aged between 7 and 17 years of both sexes, resident in all 26 states and the Federal District, found that transmission was occurring in 14 states and that large numbers of individuals were testing positive in five states, including Pernambuco. In this state, 2.14% tested positive, with local variation from 0.99 to 2.6%, and 61% of the state’s municipalities were endemic for the disease [6].

To reduce the levels of infection in communities, national control programs have adopted preventive chemotherapy involving mass drug administration (MDA) and have used diagnostic tests to monitor progress of the program and provide information to assist interventions [7]. MDA is the main strategy for controlling schistosomiasis. The aim of this strategy is to reduce morbidity and mortality resulting from the infection and avoid new infections, thus limiting transmission through reducing prevalence. The assumption is that MDA leads to a reduction in excretion of schistosome eggs and a consequent decrease in contamination of the environment and infection of the snail population, thereby reducing the size of the source of human infection [2].

In high-risk communities, where the prevalence of infection, as detected through parasitological methods, is at least 50%, all school-age children, along with at-risk adults are eligible for preventive treatment and should be treated at least once a year. Adults considered to be at risk are those who are exposed to contamination through water at work. These include individuals who work in fishing, agriculture and drainage, and women who work in the home. Whole communities living in endemic areas are also deemed eligible [1]. Communities where the prevalence is at least 10% are classified as presenting moderate risk. In these areas, school-age children and those belonging to high-risk groups should receive preventive treatment every 2 years [3].

Praziquantel is the drug of choice for mass treatment, as it is safe and cheap, thereby keeping the cost of intervention relatively low, with rapid and significant impact on the prevalence and intensity of the disease. However, even though the medication is inexpensive, the large number of people at risk of infection means that the total cost of implementation of MDA is restrictive, which makes it difficult for poor countries affected by this disease to keep mass treatment programs running on a continuous basis [8].

In the state of Pernambuco, the Sanar Program was created to tackle neglected diseases in its municipalities using strategies for controlling schistosomiasis that involved administering praziquantel to all residents for three consecutive years, in localities where 10% or more of the population tested positive in stool examination [9, 10].

The aim of the present study was to examine the effectiveness of mass treatment of *Schistosoma mansoni* infection in socially vulnerable endemic areas in the state of Pernambuco, Brazil.

## Materials and methods

### Study design

The unit of analysis in our study was the municipality. For this reason, the study design was characterized as ecological. An ecological study is an observational study defined by the level at which data are analyzed, namely at the population or group level, rather than at the individual level. The period analyzed was between the years 2011 and 2014.

### Study site and population

**Fig. 1:**
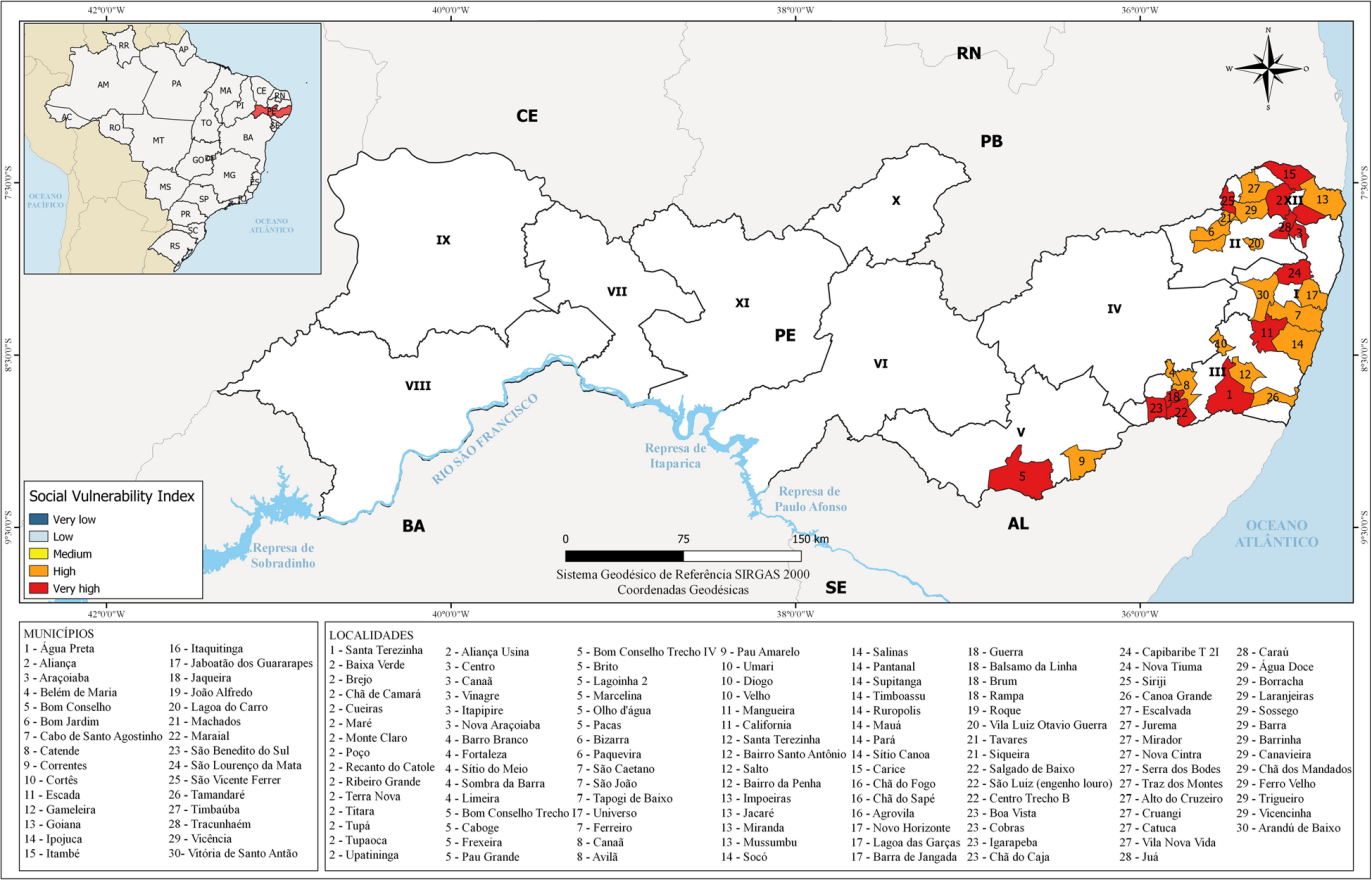
Geographical location of the state of Pernambuco, showing healthcare administration regions and the municipalities in which certain localities underwent mass drug administration, color-coded according to social vulnerability index

The state of Pernambuco is organized administratively into 12 healthcare regions. The present study covered hyperendemic localities, i.e. those in which 10% or more of the population tested positive. These localities were distributed among 30 municipalities in healthcare regions I, II, III, V and XII (Fig. 1). In relation to environmental sanitation in these localities, 71 (60.2%) did not possess access to a public water supply, 110 (93.2%) were not connected to the public sewerage network and 114 (96.6%) had no access to treatment of household waste.

The individuals living in these localities were recruited for the survey irrespective of whether or not they were taking medication. The localities were grouped according to the healthcare region to which they belonged administratively.

### Data source

The Schistosomiasis Control Program Information System (SISPCE) was used as one of the sources of data. This is a public data system in which although the possibility of missing data exists, the data consistency is as good. It is designed to monitor the epidemiological situation regarding schistosomiasis in endemic municipalities. Social and environmental data were taken from the 2010 Census conducted by the Brazilian Institute for Geography and Statistics (IBGE) and the social vulnerability index (SVI) was provided by the Institute for Applied Economic Research (IPEA).

### Mass treatment strategy

Data relating to mass treatment were gathered from the medical records filled out during the intervention. The population eligible for this action comprised all individuals living in the locality who did not present any of the following exclusion criteria: under 4 years of age; over 70 years of age; pregnant and lactating; kidney, heart and/or liver failure; convulsions; or immunosuppression [11].

In each case, the healthcare unit covering the locality was responsible for implementing treatment. Community health agents, working alongside the doctor and nurse of the unit, provided medication for the eligible population [12].

### Sample collection and examination protocols

The samples were examined in the municipal laboratory of each locality, using the Kato-Katz method, and the results were recorded in the SISPCE. The positivity rate for human infection was calculated as the number of positive cases divided by the number of samples tested, multiplied by 100. Around 4 months after the end of each mass treatment cycle, stool samples from each of the eligible localities were examined. A total of around 16,700 samples were analyzed.

This information was gathered in situ in collaboration with the community health agents of the respective localities.

Calculation of treatment coverage was based on the maximum coverage achieved in each locality in any of its treatment cycles.

### Statistical methods

#### Variables

Analysis was carried out at locality and municipality level. The variables for the localities were the following: positivity (proportion of people testing positive for schistosomiasis); zone of residence (rural or urban); and exposure to agricultural activities (cultivation and production of vegetables and fruits for human consumption), flooded areas, dump sites and leisure areas (use of rivers for leisure activities). For the municipalities, they were the locality (micro-area of the municipality) and the SVI, which included income and employment, human capital and urban infrastructure.

The SVI is produced by the IPEA. It is calculated as the arithmetic mean of 16 indicators comprising variables from the 2010 IBGE census, organized into three fields: i) urban infrastructure; ii) human capital; and iii) income and employment. The SVI is calculated using equivalent weightings for each indicator and maximum and minimum parameters, thereby creating a standardized indicator, on a normalized scale from 0 to 1, on which zero represents ideal circumstances and 1 the worst situation. The higher the index is, the worse the situation is. The results for the SVI were divided into the following strata: very low (0–0.2), low (0.2–0.3), average (0.3–0.4), high (0.4–0.5) and very high (≥ 0.5) [13].

#### Multiple correspondence analysis (MCA)

MCA is a multivariate statistical technique that enables the number of dimensions of categorical data to be reduced through using the associations that exist between the variables under study [14]. It was used here to identify groups of localities with similar patterns for categorical data, regarding exposure and zone of residence.

Application of MCA to variables generates dimensions, and these dimensions are ranked according to their contribution to the total variance of the original system. In this, the dimension that is ranked 1 accounts for the greatest proportion of the overall variance, while the other dimensions account for lower proportions. The dimensions for the MCA were chosen in such a way as to account together for around 75% of the variance.

Subsequently, the dimensions generated through MCA were used to generate clusters using the k-means method. The variables used for k-means clustering were estimates for each observation in each chosen dimension of the MCA. The elbow method was used to determine the number of clusters. The clusters were placed in order, according to mean performance, to facilitate interpretation of the model.

#### Statistical modeling

The response variable had very peculiar characteristics, which made it necessary to use a regression model capable of capturing these characteristics. For this, regression analysis was performed using the GAMLSS model (Generalized Additive Models for Location, Scale and Shape) Unlike the usual linear models, GAMLSS makes the choice of probability distribution flexible in relation to the response variable. Furthermore, it allows express modeling of all parameters of a distribution using covariables, which allows generation of more information about the phenomenon studied.

For this modeling, the probability distribution that best fitted the data of the response variable was the exponential skew power type 3, which presents four parameters: mean, variance, skewness and kurtosis. This distribution is able to model events in which the distribution presents strong asymmetry, attenuated concentration of values and light/heavy tails simultaneously.

Calculations were carried out using the R programming language version 3.6.1 for Facto Mine R version 2.0 (for MCA) and the GAMLSS software version 5.1.5 (for modeling), with a significance level of 5%.

#### Ethical approval

The present study was approved by the ethics committee of the Oswaldo Cruz University Hospital under the number CAAE 48297015.5.0000.5192.

## Results

### Characteristics of the localities

In 2011 (the baseline for the study), a population of 114,987 people underwent coproscopic examinations. The results from these examinations formed the baseline for this study. Mass treatment was performed on the entire resident population of the area, regardless of whether individuals had undergone the coproscopic examination. Out of the total of 118 locations investigated in the survey, 116 (96.3%) were found to have baseline endemicity that was considered moderate (between 10 and 50%). The lowest figure was for Lago das Garças (municipality of Jaboatão dos Guararapes) and the highest for Cobras (municipality of São Benedito do Sul). Mass treatment reached a total of 152,270 individuals. Three rounds of treatment were carried out in these localities (Table Suppl. 1).

Healthcare region XII contained 28.8% of the localities studied and, in particular, included the municipality of Aliança, which had 15 of these localities.

In most of the localities (in all healthcare regions), two rounds of MDA were conducted (44.1%). In terms of sanitary conditions, 31 (26.3%) of the localities had exposure to agricultural activities; 27 (22.9%) were exposed to streams; 33 (28%) did not have access to leisure facilities; and 96 (81.4%) were in the rural zone (Table 1).

**Table 1:** Frequency distribution of variables relating of localities that underwent mass drug administration to combat schistosomiasis, in the state of Pernambuco, Brazil, from 2011 to 2014

Variable	Number of localities	%
**Healthcare administration regions**		
I	25	21.2
II	19	16.1
III	29	24.6
V	11	9.3
XII	34	28.8
**Municipalities**		
Água Preta	1	0.8
Aliança	15	12.7
Araçoiaba	5	4.2
Belém de Maria	5	4.2
Bom Conselho	10	8.5
Bom Jardim	2	1.7
Cabo de Santo Agostinho	5	4.2
Catende	2	1.7
Correntes	1	0.8
Cortês	3	2.5
Escada	2	1.7
Gameleira	4	3.4
Goiana	4	3.4
Ipojuca	9	7.6
Itambé	1	0.8
Itaquitinga	3	2.5
Jaboatão dos Guararapes	3	2.5
Jaqueira	4	3.4
João Alfredo	1	0.8
Lagoa do Carro	1	0.8
Machados	2	1.7
Maraial	3	2.5
São Benedito do Sul	4	3.4
São Lourenço da Mata	2	1.7
São Vicente Ferrer	1	0.8
Tamandaré	1	0.8
Timbaúba	10	8.5
Tracunhaém	2	1.7
Vicência	11	9.3
Vitória de Santo Antão	1	0.8
**Number of rounds completed**		
1	28	23.7
2	52	44.1
3	38	32.2
Exposure to agricultural activities		
No	87	73.7
Yes	31	26.3
Exposure to streams and wetlands		
No	91	77.1
Yes	27	22.9
Leisure exposure		
Yes	85	72
No	33	28
Residential area		
Urban	22	18.6
Rural	96	81.4

Table 2 presents descriptive statistics for the variables included in this study. The SVI subindices for urban infrastructure, human capital and income/employment had minimum values of 0.208, 0.366 and 0.389, respectively; medians of 0.337, 0.536 and 0.573; and maximum values of 0.846, 0.76 and 0.723. The baseline endemicity index (BEI) ranged from 4.2 to 70.6, and 75% of its values were concentrated between the minimum value and 22.2%. After MDA, the BEI was found to range from 0 to 36.8, and 12.7% (*n* = 15) of the values were 0%. The mean for the endemicity index was 5.4 (range: 0 to 36.8).

**Table 2:** Descriptive statistics on the variables used in multiple correspondence analysis, in the state of Pernambuco, Brazil, from 2011 to 2014

Variable	Mean	Median	SD	Minimum	Maximum	CV (%)
Program performance	0.7	0.8	0.3	−1.1	1	43.1
Baseline endemic index	18.6	15.5	8.6	4.2	70.6	46.2
SVI	0.5	0.5	0.1	0.4	0.7	14.4
SVI urban infrastructure	0.4	0.3	0.2	0.2	0.8	40.4
SVI human capital	0.6	0.5	0.1	0.4	0.8	15.5
SVI income and work	0.6	0.6	0.1	0.4	0.7	13.4
Resident population according to locality	1290.4	326	2080.7	12	15,092	161.2
Number of households according to locality	341.9	77.5	544.9	3	3773	159.4
Post-MDA endemic index	5.4	3.7	5.7	0	36.8	107.0
% of households with water supply	32.5	0	43	0	100	132.5
% of households with sanitary installation	75.5	91.4	31.8	0	100	42.1
% of households with sewage collection	5.3	0	20.4	0	100	386.8
% of households with sewage treatment	2.5	0	13.9	0	99.6	553.9

*SD* standard deviation, *CV* Coefficient of variation

It was found that the mean proportion of households lacking a sanitary sewerage system (75.5%) was greater than the proportion lacking solid waste collection (5.3%) or water supply (32.5%). The variation coefficients indicated that there was a high degree of variation from one locality to another.

Table Suppl. 2 shows that there was no statistically significant difference in comparing the urban and rural zone, in relation to the variables of % of households with sewage collection, % of households with sewage treatment and % of households with sanitary installation. The coverage in these localities was above 85% in both zones.

### Multiple correspondence analysis (MCA)

In MCA, the variables of exposure and zone of residence were used to build up exposure clusters based on common associations for each category in these variables. Detailed results are presented in Table 3.

**Table 3:** Weights generated through multiple correspondence analysis and their dimensions, in the state of Pernambuco, Brazil, 2014

Dimensions				
**Variable**	**Categories**	**Dimension 1**	**Dimension 2**	**Dimension 3**
Exposure to agricultural activities	No	0.398	− 0.132	0.238
	Yes	−1.116	0.370	−0.668
Exposure to streams and wetlands	No	−0.409	− 0.245	− 0.024
	Yes	1.380	0.827	0.082
Exposure to evictions and waste	No	0.018	0.101	−0.187
	Yes	− 0.341	−1.892	3.490
Leisure exposure	No	−0.465	0.146	0.175
	Yes	1.198	− 0.377	− 0.451
Residential area	Urban	−0.127	1.737	0.893
	Rural	0.029	−0.398	− 0.205

**Fig. 2:**
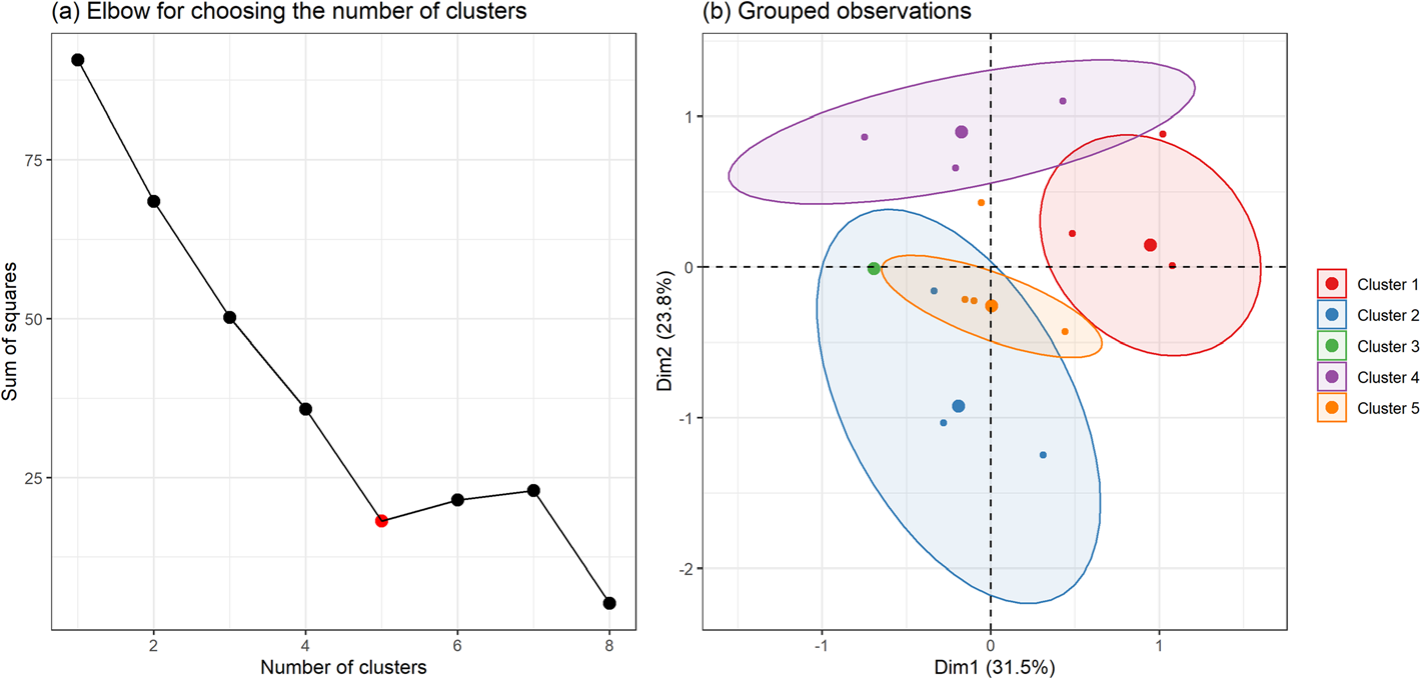
Multiple correspondence analysis map, in the state of Pernambuco, Brazil, 2014

Cluster analysis identified the formation of five clusters of localities with distinct profiles (Fig. 2 and Table 4). Cluster 1 contains 19 localities (16.1%), all with exposure to streams and wetlands, without agricultural activity or dump sites with exposed waste, predominantly in rural areas. Cluster 2 contained a smaller number of localities (*n* = 6; 5.85), characterized by no agricultural activity and no streams or wetlands but with exposed rubbish and waste. Most of these localities were rural. The 20 localities in Cluster 3 (16.9%) were all rural, with agricultural activities and access to leisure facilities but no exposure to streams and wetlands or to rubbish and waste. This was the most homogeneous cluster with regard to its characteristics. Cluster 4 comprised 19 localities (16.1%), all urban, with access to leisure facilities, mostly without exposure to rubbish and waste, exposure to streams and wetlands, or agricultural activities. Lastly, Cluster 5 contained the largest number of localities (54; 45.8%), all rural, with access to leisure facilities but no exposure to rubbish and waste.

**Table 4:** Characterization of clusters according to socio-environmental characteristics of localities, in the state of Pernambuco, Brazil, 2014

Exposure to agricultural activities	Exposure to streams and wetlands	Exposure to evictions] and waste	Leisure exposure	Residential area	Number of localities (%)	Cluster	Total (%)
No	Yes	No	Yes	Rural	4 (3.4)	1	19 (16.1)
No	Yes	No	No	Urban	2 (1.7)		
No	Yes	No	No	Rural	13 (11.0)		
No	No	Yes	Yes	Urban	1 (0.8)	2	6 (5.8)
No	No	Yes	Yes	Rural	4 (3.4)		
No	No	Yes	No	Rural	1 (0.8)		
Yes	No	No	Yes	Rural	20 (16.9)	3	20 (16.9)
No	No	No	Yes	Urban	5 (4.2)	4	19 (16.1)
No	Yes	No	Yes	Urban	7 (5.9)		
Yes	No	No	Yes	Urban	7 (5.9)		
No	No	No	Yes	Rural	36 (30.5)	5	54 (45.8)
No	No	No	No	Rural	14 (11.9)		
Yes	No	No	No	Rural	3 (2.5)		
Yes	Yes	No	Yes	Rural	1 (0.8)		

Table 5 shows the percentages of variation explained by the five MCA dimensions. The first dimension explained 31.5% of the variation and the second, 23.8%. These percentages were calculated based on a scale from 0 to 1. The dimension with greatest variation presented variation of an order of magnitude higher than its own value. The two first dimensions explained 55.3% of the variance, while none of the remaining dimensions explained more than 21.5%.

**Table 5:** Percentages of variation explained by the five multiple correspondence analysis dimensions, in the state of Pernambuco, Brazil, 2014

Dimension	Eigenvalue	% variance	Cumulative % variance
1	0.3	31.5	31.5
2	0.2	23.8	55.3
3	0.2	21.5	76.8
4	0.1	13.5	90.4
5	0.1	9.6	100.0

**Fig. 3:**
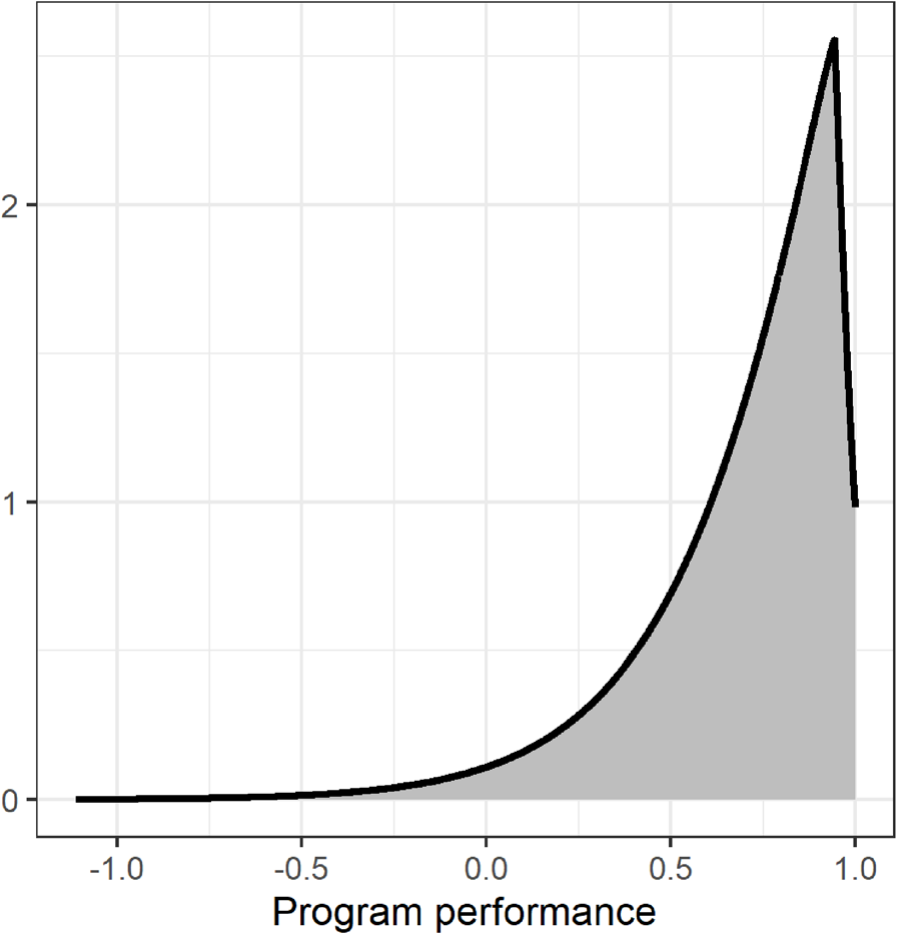
Estimated distribution of mass drug administration effectiveness, in the state of Pernambuco, Brazil, 2014

The parameters estimated for the effectiveness of MDA were the following: mean = 0.94394 (*p* < 0.001); variance = 0.07964 (*p* < 0.001); skewness = 0.40543 (*p* < 0.001); and kurtosis = 1.17128 (*p* = 0.5). Kurtosis had a high *p* value, equivalent to 1. The estimate provided by the model was used to plot the curve in Fig. 3.

**Fig. 4:**
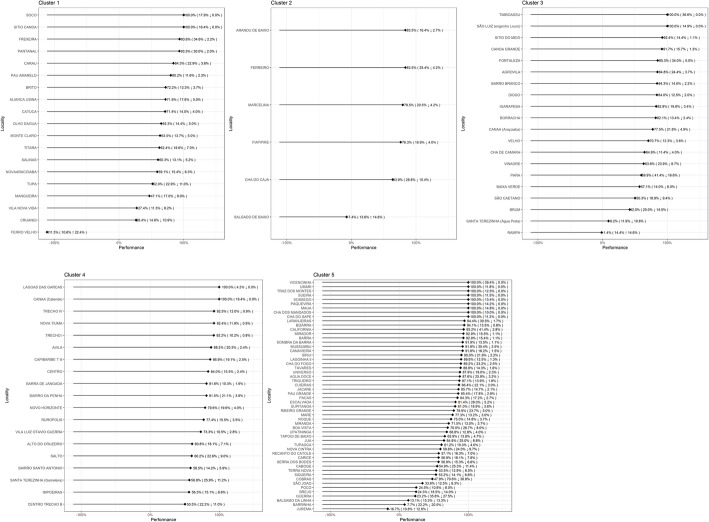
Clusters of mass drug administration effectiveness in each locality, with the initial and final endemic levels, in the state of Pernambuco, Brazil, 2014

Some localities presented negative effectiveness. This means that there was an increase in the endemicity index when the probability of this occurring was less than 3%. These can therefore be considered atypical occurrences. The localities falling into this category were Rampa (− 1.39%); Salgado de Baixo (− 7.35%); Jurema (− 16.7%); and Ferro Velho (− 111%). The probability of achieving effectiveness superior to 50% was 76.5% (Fig. 4). The effectiveness of each locality is presented in Fig. 4. Each line represents effectiveness, while the first number in parentheses is the baseline endemicity and the second, the endemicity index after mass treatment. Successful localities are marked by an arrow pointing down, and unsuccessful ones by an arrow pointing upwards.

Table 6 shows the estimates for the parameters of the model that was used to generate the results. Effectiveness increased with the number of rounds of treatment conducted. Other than this, the effect on the mean changed in accordance with the baseline endemicity index.

**Table 6:** Parameters of the model used to generate the results, in the state of Pernambuco, Brazil, 2014

**Parameter**	**Coefficient**	**Estimate**	***P*-value**
μ	Intercept	1.1454	0
	Number of rounds done: 2	0.1362	0
	Number of rounds done: 3	0.1386	0
	Baseline endemic index	0.0141	0
	SVI income and work	−0.0395	0
	Cluster 2	−0.1167	0
	Cluster 3	0.0751	0
	Cluster 4	0.2575	0
	Cluster 5	0.1839	0
	(Number of rounds done: 2) * (Baseline endemic index)	0.0003	0
	(Number of rounds done: 3) * (Baseline endemic index)	−0.0006	0
	(Cluster 2) * (Baseline endemic index)	−0.0027	0
	(Cluster 3) * (Baseline endemic index)	−0.0051	0
	(Cluster 4) * (Baseline endemic index)	−0.0119	0
	(Cluster 5) * (Baseline endemic index)	0	0.98
	(Baseline endemic index) * (SVI human capital)	−0.0251	0
log(σ^2^)	Intercept	−17.539	0
	Cluster 2	−12.191	0
	Cluster 3	−13.025	0
	Cluster 4	14.5965	0
	Cluster 5	14.8149	0
log(ν)	Intercept	−0.911	0
log(τ)	Intercept	−1.7454	0
	Cluster 2	−0.2965	0
	Cluster 3	−0.4099	0
	Cluster 4	2.1373	0
	Cluster 5	1.8407	0
**Random Effects**			
**Parameter**	**Municipalities**	**Beta 00**	
μ	Itaquitinga	−0.23	
	Jaboatão dos Guararapes	−0.48	
	Jaqueira	−0.71	
	João Alfredo	−0.64	
	Lagoa do Carro	−0.61	
	Machados	−0.5	
	Maraial	−0.07	
	São Benedito do Sul	−0.26	
	São Lourenço da Mata	−0.48	
	São Vicente Ferrer	−0.43	
	Tamandaré	−0.32	
	Timbaúba	−0.58	
	Tracunhaém	−0.43	
	Vicência	−0.46	
	Vitória de Santo Antão	−0.29	
	Água Preta	−1.15	
	Aliança	−0.49	
	Araçoiaba	−0.21	
	Belém de Maria	−0.35	
	Bom Conselho	−0.24	
	Bom Jardim	−0.39	
	Cabo de Santo Agostinho	−0.18	
	Catende	−0.21	
	Correntes	−0.43	
	Cortês	−0.41	
	Escada	−0.51	
	Gameleira	−0.49	
	Goiana	−0.61	
	Ipojuca	−0.21	
	Itambé	−0.74	

For two rounds, for every extra 1% of initial endemicity index, the fixed effect (for BEI = 0) of 13.62% increased by 0.0003% and reached a maximum of 15.94% (when the baseline endemic index, BEI, also reached its maximum). When the number of rounds was 3, the fixed effect was 13.86% and this diminished by 0.0006% for every extra 1% of initial endemicity index and could fall to as low as 9.58%.

The clusters also interacted with the BEI, with the exception of Cluster 5 (*p* = 0.9843). Cluster 2 always had negative effects, with reductions increasing by 0.273% for every 1% of BEI. For Cluster 3, the effect on performance was positive for values less than 14.81%, such that each 1% of BEI was associated with a reduction of around 0.508%. Cluster 4 exhibits a similar pattern to that of Cluster 3, with a positive effect for values lower than 21.58%, such that the effect diminished by around 1.19% for every further 1% of BEI. Cluster 5 showed a positive effect of 18.39% on performance regardless of BEI, which had no effect on the outcome.

The human capital component of the SVI showed an association with BEI. The marginal effect of SVI human capital was always negative, decreasing by 0.025% for every 1% increase in BEI. The effects caused by this variable for this sample ranged from − 34.8% to − 3.86%.

**Fig. 5:**
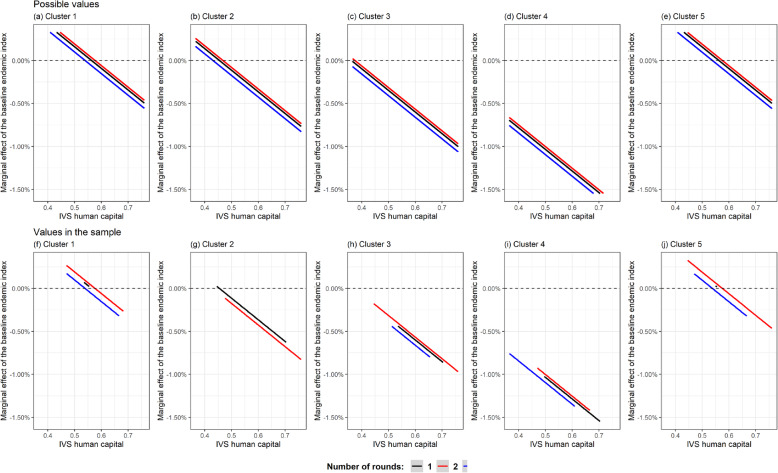
Marginal effect of the baseline endemic index for different clusters, in the state of Pernambuco, Brazil, 2014

The effect of BEI (Fig. 5) on the mean could be positive or negative, depending on the SVI subindex for human capital, the number of rounds and the cluster to which the locality belonged.

The marginal effect of BEI could only be positive in Clusters 1, 2 and 5. This was seen only in the following cases for Clusters 1 and 5: SVI human capital < 0.562 (for one round); SVI human capital < 0.573 (for two rounds); and SVI human capital < 0.538 (for 3 rounds). It was also seen in the following cases for Cluster 2: SVI human capital < 0.454 (for one round); SVI human capital < 0.466 (for two rounds); and SVI human capital < 0.538 (for three rounds). For Cluster 3, there was a very slight possibility that the effect of BEI would be positive, while the likelihood of this for Cluster 4 was zero.

For the sample as a whole, in 46.6% (*n* = 55) of the localities, the effect of the BEI was positive, ranging from 0 to 9.54%. The other values were negative and ranged down to as low as − 34.34% (Fig. 5).

The income component of the SVI did not appear to interact with any variable. Its effect was therefore fixed. For every 0.01 of additional SVI income, program effectiveness diminished by 0.0395%. The more socially vulnerable the locality was, in terms of income and employment, the poorer the effectiveness of the strategy also was.

The effect of the random intercepts on the mean also needed to be considered. These effects could be understood as the extent to which the reference effect on performance (estimate of overall intercept = 1.1454) was affected in each municipality overall. All the intercepts were negative and it should be noted that some municipalities had very similar estimates, as in the case of Jaboatão dos Guararapes, São Lourenço da Mata, Vicência, Aliança and Gameleira.

## Discussion

The results showed that the mass treatment intervention in the localities studied brought about a significant reduction in the numbers of individuals testing positive for schistosomiasis. This concurs with results obtained using this strategy in Kenya, where two treatment cycles reduced infection by 65%. In Uganda, the figure was 70% and, in Sierra Leone, 3 cycles led to a 67% reduction [15–17]. This suggests that mass drug administration may have an impact in terms of reducing the parasite burden in the environment, thus diminishing transmission of the disease.

The number of treatment cycles contributed towards reducing the positivity rate in the localities. The larger the number of cycles carried out, the greater the reduction was. Likewise, more extensive treatment coverage may also have contributed to greater reductions in positivity. The same occurred in Sierra Leone, Kenya and Uganda, where at least two treatment cycles were conducted, with significant reductions in positivity [15, 16].

Multiple correspondence analysis further showed that social and environmental factors and social vulnerability also influenced the effectiveness of MDA.

Analysis on epidemiological data, the degree of coverage of actions combating the disease (degree of coverage of MDA) and social and environmental factors, were all important for establishing which areas should have priority, with regard to planning and supervision in the state of Pernambuco [18].

The present study revealed that reductions in the numbers of individuals testing positive for schistosomiasis were achieved, although social and environmental conditions remain poor. In a previous study [19], cycles of treatment of schistosomiasis in the state of Pernambuco were assessed and reductions in prevalence were found. It was noted in that study, however, that the paucity of environmental changes ruled out the possibility that environmental factors had contributed to this outcome. A control program based solely on administration of praziquantel is ineffective and unsustainable over the long term. To achieve interruption of transmission, additional strategies need to be adopted in combination with preventive drug treatment [19].

The data revealed considerable declines in prevalence, from the baseline to the final tests, despite a lack of consistency in the numbers of individuals examined and the numbers of rounds of MDA in some localities. Prior to the intervention, these localities presented positivity rates above the recommended level (5%) and were thus characterized as areas at high risk of transmission of schistosomiasis. After treatment, the rates dropped, thus ensuring that positivity came within acceptable levels in various localities.

It was found that increasing the number of rounds always improved performance. However, the effect of two rounds increased in proportion to the initial endemicity index, while the effect of three rounds diminished. The number of rounds made little difference for lower initial endemicity indices. In most cases, therefore, there is a need to examine the costs and benefits of carrying out two or three rounds.

Thus, despite the variation in treatment coverage among both communities and age groups, mean coverage provides a reasonable estimate of the mean effect of the parasite burden on the population [10, 13].

In addition to the number of cycles implemented, treatment coverage was also found to be an important factor in reducing positivity. Analysis on the coverage achieved by each treatment cycle in the present study showed that 72% of the localities obtained good results from adherence to the strategy, with coverage of 75% or more of the eligible population.

The number of treatment cycles was one factor that contributed to the magnitude of the effect observed. This turned hyperendemic areas into areas of low endemicity and demonstrated a capacity to reduce the morbidity and mortality caused by schistosomiasis in vulnerable communities.

This study used MCA, which is a kind of exploratory factor analysis that aims to reduce the number of dimensions in large groups of categorical data, in order to identify social and environmental factors and social vulnerability as factors associated with the effectiveness of MDA in the localities surveyed. This multivariate statistical technique has the advantage of being able to extract significant quantities of information using fewer dimensions. Associations with the effectiveness of MDA can be visualized in terms of the similarities and differences between localities. Furthermore, each locality can be interpreted using dimensions based on graphs of the behavioral categories of the variables. This technique also enables use of pre-existing datasets, which in the present study comprised data from the IBGE, IPEA and SISPCE.

MCA identified five main indicators relating to the effectiveness of MDA, and three dimensions. A model of the variables involved in the dynamics of the epidemic may help to develop more clearly directed and more effective actions in control programs.

Five clusters of localities with distinct profiles were identified. It was found that the larger the cluster number was, the greater the likelihood of improved MDA effectiveness also was. For example, localities in Cluster 1 did not benefit much, while all of those in Cluster 5 did benefit.

Cluster 1, containing mostly rural localities and those with exposure to streams and wetlands, did not show enhanced effectiveness of MDA. Flooded land and wetlands where humans frequently come into contact with snail habitats are highly conducive to transmission of schistosomiasis [20, 21]. Consequently, the most significant determining factors are the patterns of human contact with water, levels of sanitation and hygiene and abundance and susceptibility of freshwater snail hosts [22]. A study in Ethiopia on the effects of water sources, sanitation and hygiene on the prevalence of *Schistosoma mansoni* infection among school children found that lack of clean water for bathing, washing and swimming and poor sanitation and hygiene were the main risk factors [23].

The human capital and income/employment components of the SVI had negative effects on performance. Human capital, which involves indicators relating to health conditions and access to education, was the explanatory variable that had the greatest marginal effect on the performance of MDA. Socially vulnerable people are the one who are most affected by parasitic infections caused by intestinal helminths and protozoa [24]. Research on schistosomiasis in Pernambuco has shown that socioeconomic conditions are strongly associated with its occurrence [19]. It is well known that, as a disease of poverty, schistosomiasis is both a cause and a consequence of poor socioeconomic conditions in endemic areas [25].

The elimination of schistosomiasis as a public health problem and interruption of transmission have been established by the World Health Organization (WHO) as goals for 2025. Schistosomiasis is a localized disease and interruption of transmission and elimination of this disease as a public health problem thus require strategies adapted to the micro-epidemiology and local culture [26]. MDA alone substantially reduces the prevalence but not enough to interrupt transmission. An approach that reduces or prevents contamination of bodies of freshwater and hence halts transmission of the disease needs to involve not only MDA but also to include improvements in access to water, sanitation and hygiene (WASH), and changes in behavior [26–29].

### Limitations

The present study had a number of limitations: i) the secondary data from the SISPCE may have been incomplete since this is a public data system in which the possibility of missing data exists; nonetheless, its data consistency is good; ii) fluctuations in the numbers of individuals covered by studies in these localities over the years may have led to underestimation of prevalence; iii) the Kato-Katz technique used in the program and recommended by the WHO is less sensitive in areas with low and moderate prevalence; and iv) the ecological fallacy, i.e. the belief that relationships observed for groups necessarily hold for individuals. In our paper, in the description and discussion of the results, we always refer to effects relating to clusters, which in our case were the localities. Regarding the differences between the localities studied, the localities selected were those in which 10% or more of the population had tested positive. For this reason, clusters that were homogenous in relation to social and economic conditions, as explained by the disease studied, i.e. *Schistosoma mansoni* infection, were more likely to be seen under unfavorable health conditions and in poor areas. The effect of urban and rural variables on the results was not important because, even though most of the localities were in rural areas, the exposure was similar to that of the localities in urban areas, as homogenized through the selection criteria for these areas studied. Despite these limitations, the present study has provided results that may help to develop schistosomiasis surveillance and control actions.

## Conclusion

MDA gave rise to reductions in the endemic index when performed in up to two rounds. This information is useful for control and elimination actions in endemic areas. We recommend that, in addition to MDA, it should be accompanied by basic sanitation actions, monitoring of intermediate vectors and case surveillance. The MCA methodology proved to be useful in identifying the distribution of social and environmental conditions and social vulnerability associated with the effectiveness of MDA for combating schistosomiasis.

## Supplementary Information

**Additional file 1: Table Suppl 1.** Distribution of locations submitted to mass drug administration for schistosomiasis with number of MDA rounds, proportion of positive cases and coverage per year, the state of Pernambuco, Brazil, from 2011 to 2014.

**Additional file 2: Table Suppl 2.** Descriptive statistics on the socioeconomic variables of urban and rural areas, the state of Pernambuco, Brazil, from 2011 to 2014.

## Data Availability

The datasets used and/or analyzed during the current study are available from the corresponding author upon reasonable request.

## Abbreviations

BEI: Baseline endemicity index
IBGE: Brazilian Institute of Geography and Statistics
IPEA: Institute for Applied Economic Research
MCA: Multiple correspondence analysis
MDA: Mass drug administration
NTDs: Neglected tropical diseases
SISPCE: Schistosomiasis Control Program Information System
SVI: Social vulnerability index
WASH: Water, sanitation and hygiene
