# Supplementary material for: Effectiveness of mass treatment of Schistosoma mansoni infection in socially vulnerable areas of a state in northeastern Brazil, 2011–2014
Source: Arch Public Health. 2021 Mar 9;79:30. doi: 10.1186/s13690-021-00549-9 (PMC7941929; doi:10.1186/s13690-021-00549-9)
Supplement: Supplementary file 1 — Additional file 1: Table Suppl 1. Distribution of locations submitted to mass drug administration for schistosomiasis with number of MDA rounds, proportion of positive cases and coverage per year, the state of Pernambuco, Brazil, from 2011 to 2014. [file 13690_2021_549_MOESM1_ESM.pdf]

Supplementary Information

Table Suppl 1 Distribution of locations submitted to mass drug administration for schistosomiasis with number of MDA rounds, proportion of positive cases and coverage per year, the state of Pernambuco, Brazil, from 2011 to 2014

Municipality Locations	2011- Baseline			2012				2013				2014			
	**POP**	**IP**	**ROU**	**POP**	**EX**	**IP**	**COB1**	**POP**	**EX**	**IP**	**COB2**	**POP**	**EX**	**IP**	**COB3**
I Health Region															
**Jaboatão dos Guararapes**															
Barra de Jangada	3,384	10.3	3	3,384	410	1.9	81.6	2,051	168	0.6	92.3	3,869	410	1.9	84.4
Lagoa das Garças (piloto)	1,844	4.2	3	1,844	400	0	81.9	1,532	404	0.7	97.7	1,354	100	0	83.6
Novo Horizonte	3,163	19.6	3	3,163	417	4	89.6	2,464	109	1.8	82	2,513	417	4	81.1
**Araçoiaba**															
Centro	3,179	15.3	1	3,179	245	2.4	79	-	-	-		-	-	-	
Canaã	562	21.8	1	562	143	4.9	79.5	-	-	-		-	-	-	
Vinagre	270	23.9	1	270	23	8.7	58.5	-	-	-		-	-	-	

Itapipiré	500	16.9	1	500	124	4	78.6	-	-	-		-	-	-	
Nova Araçoiaba	1,701	15.4	1	1,701	79	6.3	75.8	-	-	-		-	-	-	
**Cabo de Santo Agostinho**															
Ferreiros (Mercês)	680	25.4	2	680	-	-	77.4	-	-	-		680	142	4.2	
São Caetano	124	18.9	2	124	-	-	92.5	-	-	-	100	104	64	9.4	
São João	100	12.5	2	100	-	-	96.7	-	-	-	90.2	121	48	8.3	
Tapugi de Baixo	681	13.8	2	681	231	4.7	97.6	-	-	-	99.8	412	231	4.7	
Universo	77	19	2	77	-	-	100	-	-	-	78.3	85	43	2.3	
**Ipojuca**															
Mauá	52	14.8		-	-	-	40.1	-	-	-	100	46	3	0	
Pantanal	1,345	30	2	-	-	-	83.7	-	-	-	65.7	474	45	2.2	
Pará	211	41.4	3	-	-	-	80.4	-	-	-	43.4	113	12	16.6	74
Ruropólis	5,811	15.5	3	-	-	-		-	-	-	16	1,099	288	3.5	98
Salinas	2,428	13.1	3	-	-	-		-	-	-	23.8	1,068	173	5.2	75
Sítio canoa	480	16.4	3	-	-	-		-	-	-	48.2	338	21	0	68

Socó	1,078	17.9	3	-	-	-		-	-	-	32.3	487	55	0	100
Supitanga	445	18.9	3	-	-	-		-	-	-	15.1	119	28	3.6	
Timboassú	120	39.6	3	-	-	-		-	-	-	71.6	202	9	0	
São Lourenço da Mata															
Nova Tiúma	4,599	10	3	4,599	304	0.98	87.5	5,228	340	2.3	87.7	2,536	250	5.6	96.8
Capibaribe – trecho III (Várzea Fria e Umuarama)	11,074	19	3	11,074	-	-	80	9,276	257	5.1	67.8	-	79	2.5	63.9
**Vitória de Santo Antão**															
Arandu de Baixo	237	16	2	237	134	2,72	87.7	207	8	0	93.7	-	-	-	83.8
II Health Region															
**Bom Jardim**															
Bizarra	3,331	13.5	2	3,331	236	0,8	69.2	-	-	-	100	3,039	976	2.2	
Paquevira	680	14.2	2	680	154	0	81.2	-	-	-	51.2	695	183	1.1	
**João Alfredo**															
Roque	1,42	14.8	2	1,42	261	3.7	86.7	-	-	-	75.1	-	-	-	

**Lagoa do Carro**															
Vila Luiz Otávio Guerra	840	10.5	2	840	-	-	46	988	170	4.1	28.5	-	36	2.8	
**Machados**															
Tavares	320	14.3	2	-	-	-	69	-	-	-	76.7	247	63	1.6	
Siqueira	420	14.1	2	-	-	-	69.4	-	-	-	86.7	211	75	6.6	
**Tracunhaém**															
Juá	40	25	3	40	45	8.8	94.6	39	32	6.2	94.9	-	-	-	76.1
Caraú	162	22.9	3	162	136	3.7	92.2	134	116	1.7	75.4	-	-	-	61.5
**Vicência**															
Água Doce	74	25.9	1	74	53	0	83.6	-	-	-	41.3	-	-	-	
Borracha	2,652	13.4	1	2,652	136	-	49.5	-	-	-	69.2	-	-	-	
Laranjeiras	1,03	30.5	1	1,03	234	1.7	75.9	-	-	-	67.6	-	-	-	
Sossego	231	13.4	1	231	78	0	70.6	-	-	-	73.7	-	-	-	
Barra	663	15.4	1	663	174	1.1	80.1	-	-	-	35.4	-	-	-	
Barrinha	156	22.2	1	156	39	20.5	82.2	-	-	-	72.6	-	-	-	

Canavieira	106	18.1	1	106	68	1.5	78.3	-	-	-	31.3	-	-	-
Chã dos Mandados	60	10	1	60	94	0	77.6	-	-	-	29.6	-	-	-
Ferro Velho	28	10.6	1	28	58	22.4	78.8	-	-	-	60.8	-	-	-
Trigueiro	1,58	13.9	1	1,58	110	1.8	92.8	-	-	-	71	-	-	-
Vicencinha	70	39.4	1	70	39	0	79.2	-	-	-	72.4	-	-	-
V Health Region														
**Bom Conselho**														
Bom Conselho trecho I	1,509	10.2	2	1,509	124	0.8	46.6	-	-	-	34.1	-	-	-
Cabogé	120	25.3	2	120	61	11.4	75	-	-	-	100	-	-	-
Frexeira	50	34.6	2	50	44	2.2	76	-	-	-	100	-	-	-
Pau Grande	61	17.8	2	61	38	2.6	83.6	-	-	-	100	-	-	-
Bom Conselho trecho IV	6,186	12	2	6,186	223	0.9	48.6	-	-	-	99.6	-	-	-
Brito	235	13.3	2	235	80	3.7	66.4	-	-	-	100	-	-	-
Lagoinha II	130	12.5	2	130	75	1.3	82.6	-	-	-	100	-	-	-
Olho d'água	15	14.4	2	15	59	5	62.2	-	-	-	100	-	-	-

Marcelina	90	20.5	2	90	119	4.2		-	-	-	100	-	-	-	
Pacas	68	17.2	2	68	36	2.7	51.5	-	-	-		-	-	-	
**Correntes**															
Pau Amarelo	515	7.6	2	515	262	2.3	69.4	-	-	-	49.7	-	-	-	
III Health Region															
**Água Preta**															
Eng. Sta Terezinha e Usina Sta Terezinha	1,505	11.9	2	1,505	184	10.8	66.4	1,393	119	0	44	1,264	110	0.9	44
**Belém de Maria**															
Barro Branco	117	14	3	117	99	2	72.2	81	79	0	69.1	-	-	-	50.9
Fortaleza	102	343	3	102	60	5	50.6	25	31	0	80	-	-	-	35.9
Sítio do Meio	229	14.4	3	229	177	1.1	62.4	172	114	0	91.3	-	-	-	69
Sombra da Barra	361	13.5	3	361	331	1,1	78	265	244	1,6	65.8	-	-	-	46.1
**Catende**															
Canãa	3,783	19.4	2	3,783	188	0	93.5	-	-	-	75.3	-	-	-	42.5

Avilã	1,66	20.3	2	1,66	205	2.4	33	-	-	-	87.5	-	-	-	75.5
**Cortes**															
Umari	228	11.8	2	228	45	0	90.8	125	72	0	90.4	-	-	-	96.4
Diogo	99	12.5	2	99	49	2	87.2	100	75	2	85	-	-	-	96.9
Velho	396	12.3	2	396	110	3.6	98.8	169	28	0	91.1	-	-	-	63.1
**Escada**															
Mangueira	3,099	17	2	3099	364	9	65.6	-	-	-	59.4	-	-	-	
Califórnia	159	41.4	2	159	145	2.8	90.5	-	-	-		-	-	-	
**Gameleira**															
Salto/Santa Maria	562	25.9	2	562	265	9.1	77.2	-	-	-		-	-	-	
Bairro da Penha	454	14.2	2	454	178	3.9	86	-	-	-		-	-	-	
Santa Terezinha	354	22.6	2	354	285	11.2	33.2	-	-	-		-	-	-	
Bairro Santo Antonio / Santa Luzia	921	21.1	2	921	285	5.9	95.3	-	-	-		-	-	-	
**Jaqueira**															
Guerra	227	19	2	227	120	17.5	64.8	69	13	15.4	89.8	143	100	12.0	86

Rampa	227	18.7	2	227	109	4.6	64.8	69	2	0	89.8	143	100	12.0	86
Balsamo da Linha	197	20.3	2	197	150	13.3	86.2	132	11	9.1	61.4	229	58	6.9	62
Brum	197	20.3	2	197	124	14.5	86.2	132	8	12.5	61.4	229	58	6.9	62
**Maraial**															
Salgado de Baixo	897	13.6	2	897	82	14.6	51.8	82	4	0		-	-	-	
Engenho Louro	74	15	2	74	21	0	92.5	21	2	0		-	-	-	
Centro trecho B	2,296	22.2	2	2,296	173	11	42.8	173	203	2.9		-	-	-	
**São Benedito do Sul**															
Boa Vista	956	27	2	956	100	8	88.7	676	121	0.8	67.8	143	-	-	
Cobras	80	70.6	2	80	68	36.8	95.9	70	30	6.7	80	88	-	-	
Igarapeba	1,142	19.8	2	1,142	116	3.4	90.7	889	129	4.6	76.7	937	-	-	
Chã do Cajá	438	29	3	438	96	10.4	96.1	692	55	5.4	78.8	391	-	-	90
**Tamandaré**															
Canoa Grande	212	16	3	212	159	1,3	80.4	127	154	0	80.3	-	-	-	76.5
XII Health Region															

**Aliança**													
Recanto do Catolé	409	16.3	1	409	161	7	84	-	-	-	-	-	-
Tupaoca	955	10.3	1	955	148	4	85	-	-	-	-	-	-
Cuieiras	72	22.1	1	72	66	3	85	-	-	-	-	-	-
Maré	167	13.2	1	167	107	3	84	-	-	-	-	-	-
Baixa Verde	155	14	1	155	77	6	71	-	-	-	-	-	-
Poço	165	10.6	1	165	109	8	73	-	-	-	-	-	-
Brejo	45	18.5	1	45	90	14	76	-	-	-	-	-	-
Ribeiro Grande	74	23.7	1	74	67	5	84	-	-	-	-	-	-
Terra Nova	122	13	1	122	82	6	93	-	-	-	-	-	-
Titara	79	19	1	79	53	7	70	-	-	-	-	-	-
Monte Claro	103	13.6	1	103	90	5	79	-	-	-	-	-	-
Tupã	72	22.9	1	72	65	11	62.3	-	-	-	-	-	-
Chã de Camará	159	11.4	1	159	6	4	80.5	-	-	-	-	-	-
Upatininga	2,516	12.8	1	2,516	152	4	79	-	-	-	-	-	-

Usina Aliança	331	17.8	1	331	118	5	63.1	-	-	-	71	-	-	-	
**Goiana**															
Jacaré	76	14.7	2	76	106	2.1	76.3	-	-	-	21.2	-	-	-	
Miranda	48	13	2	48	37	3.7	70.7	-	-	-	92.8	-	-	-	
Mussumbú	214	30	2	214	155	2.6	74.0	-	-	-	63.2	-	-	-	
Impoeiras	509	15.1	2	509	167	6.6	79.6	-	-	-	38.8	-	-	-	100
**Itambé**															
Caricé	2,431	18.1	2	2,431	230	7.82	73.2	1,141	110	3.4	48.5	-	-	-	
**Itaquitinga**															
Chã do Fogo	2,355	23.2	1	2,355	160	2.5	62	-	-	-		-	-	-	
Chã do Sapé	1,71	11.2	1	1,71	121	0	70	-	-	-		-	-	-	
Agrovila (Itaquitinga)	3,184	24.4	1	3,184	163	3.7	67	-	-	-		-	-	-	
**São Vicente Ferrer**															
Siriji	4,345	21.9	2	4,345	224	2.2	74.6	3,876	1,177	2.2	90	-	-	-	
**Timbaúba**															

Alto do Cruzeiro	74	25.9	2	74	-	-	46	-	115	2,6	82	-	-	-
Cruangi	2,652	13.4	1	2,652	-	-	89	-	-	-	89	-	57	0
Catucá	1,03	30.5	1	1,03	-	-	66	25	1	-	66	-	25	4
Escalvada	159	13.4	2	159	-	-	85	76	53	-	78	-	13	0
Jurema	663	15.4	2	663	-	-	85.6	87	126	-	83	-	5	0
Mirador	156	22.2	1	156	-	-	82.5	-	-	-	92	51	7	0
Nova Cintra	106	18.1	2	106	-	-	84.4	41	97	-	69	-	5	20
Serra dos Bodes	171	10	2	171	-	-	92.6	90	66	-	84	-	9	0
Traz dos Montes	51	10.6	2	51	-	-	83,3	30	0	-	72	-	3	0
Vila NovaVida	1,58	13.9	2	1,58	-	-	69	38	105	-		-	-	-

END= Endemicity after the MDA 1-Low: < 10% 2- Moderate: ≥ 10% a ≤50% 3-High: > 50%. POP= Population in the locality. IP = Proportion of positive cases. ROU = Number of MDA rounds in which the locality was submitted. EX = Number of exams performed. COB1 = Coverage of treatment in the 1st round. COB2 = Coverage of treatment in the 2st round. COB3 = Coverage of treatment in the 3st round
