# Supplementary material for: Effectiveness of mass treatment of Schistosoma mansoni infection in socially vulnerable areas of a state in northeastern Brazil, 2011–2014
Source: Arch Public Health. 2021 Mar 9;79:30. doi: 10.1186/s13690-021-00549-9 (PMC7941929; doi:10.1186/s13690-021-00549-9)
Supplement: Supplementary file 2 — Additional file 2: Table Suppl 2. Descriptive statistics on the socioeconomic variables of urban and rural areas, the state of Pernambuco, Brazil, from 2011 to 2014. [file 13690_2021_549_MOESM2_ESM.docx]

**Table Suppl 2** Descriptive statistics on the socioeconomic variables of urban and rural areas, the state of Pernambuco, Brazil, from 2011 to 2014

| **Variable** | **Urban zone**  **Median (IQR)** | **Rural zone**  **Median (IQR)** | **p-value** |
| --- | --- | --- | --- |
| **% of households with sanitary installation** | 98.9 (90.0 – 100) | 86.2 (44.8 – 99.3) | 0.002 |
| **% of households with sewage collection** | 0 (0 – 0) | 0 (0 – 0) | 0.532 |
| **% of households with sewage treatment** | 0 (0 – 0) | 0 (0 – 0) | 0.360 |
| **Baseline endemic index (%)** | 15.5 (11.9 – 19.6) | 15.6 (13.4 – 23.0) | 0.290 |
